# Pulmonary Hamartoma Initially Misdiagnosed as Adenocarcinoma: A Case Report and Review of the Literature

**DOI:** 10.1002/rcr2.70445

**Published:** 2025-12-17

**Authors:** Erfaneh Hosseini, Maryam Mazraehei Farahani, Ramin Sami, Mohammad Behgam Shadmehr

**Affiliations:** ^1^ Department of Internal Medicine, School of Medicine Isfahan University of Medical Science Isfahan Iran; ^2^ Farhikhtegan Medical Convergence Sciences Research Center, Farhikhtegan Hospital Tehran Medical Sciences Islamic Azad University Tehran Iran; ^3^ Tracheal Diseases Research Center, National Research Institute of Tuberculosis and Lung Diseases (NRITLD) Shahid Beheshti University of Medical Sciences Tehran Iran

**Keywords:** diagnosis, hemoptysis, lung neoplasm, pulmonary hamartoma, video‐assisted thoracoscopic surgery

## Abstract

Pulmonary hamartomas are common benign lung tumours that rarely present with hemoptysis and may mimic malignancy. We report a 39‐year‐old non‐smoker with 2 months of hemoptysis and a 3‐cm right upper‐lobe lesion. CT‐guided core biopsy with IHC suggested adenocarcinoma (CK7, TTF‐1, Napsin‐A positive), but PET‐CT showed no metastasis. The patient underwent VATS resection and final histopathology confirmed pulmonary hamartoma. Symptoms resolved postoperatively with no recurrence at 6‐month follow‐up. This case highlights potential false‐positive biopsy/IHC results from entrapped benign epithelium and the role of surgical excision for definitive diagnosis.

## Graphical abstract

A pulmonary hamartoma mimicking adenocarcinoma on biopsy. The case emphasises the importance of surgical excision when histopathological and imaging findings are discordant.

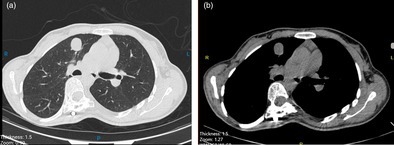

## 1 Introduction

Pulmonary hamartomas (PHs) are benign neoplasms composed of mature but disorganised mesenchymal tissue elements, including cartilage, fat, and fibromyxoid stroma. They constitute approximately 6%–8% of solitary pulmonary nodules and are the most frequent benign tumours of the lung. Radiologically, PHs typically manifest as well‐circumscribed pulmonary nodules occasionally demonstrating pathognomonic fat density or characteristic ‘popcorn’ calcifications, aiding differentiation from malignant neoplasms. However, in the absence of these classic imaging hallmarks, clinically and radiologically may be diagnosed incorrectly [1, 2, 3].

Although most PHs are asymptomatic incidental findings, symptomatic presentations such as hemoptysis are rare and usually raise suspicion for malignancy or endobronchial pathology. Diagnostic reliance on small biopsy specimens and immunohistochemical (IHC) analyses may lead to misinterpretations, especially due to sampling errors or nonspecific staining of entrapped benign epithelium. Herein, we describe a case of pulmonary hamartoma in a young female initially misdiagnosed as adenocarcinoma on needle biopsy, highlighting diagnostic pitfalls and emphasising the crucial role of surgical excision for definitive diagnosis [4, 5].

## 2 Case Report

A 39‐year‐old woman presented with a two‐month history of persistent cough and haemoptysis.

**FIGURE 1:**
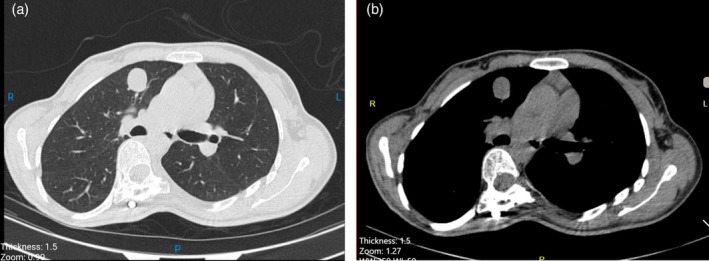
(a) Axial chest CT (parenchymal window) showing a well‐circumscribed 3 cm round lesion in the right upper lobe. (b) Mediastinal window of the same lesion showing no visible fat density or popcorn calcification.

Initial chest computed tomography (CT) revealed a 3‐cm round lesion located in the right upper lobe, raising suspicion for a pulmonary neoplasm and prompting further diagnostic evaluation (Figure 1).

Subsequently, bronchoscopy was performed to inspect the airways directly. It revealed blood‐stained tracheal mucosa, but no visible intraluminal mass was detected.

**FIGURE 2:**
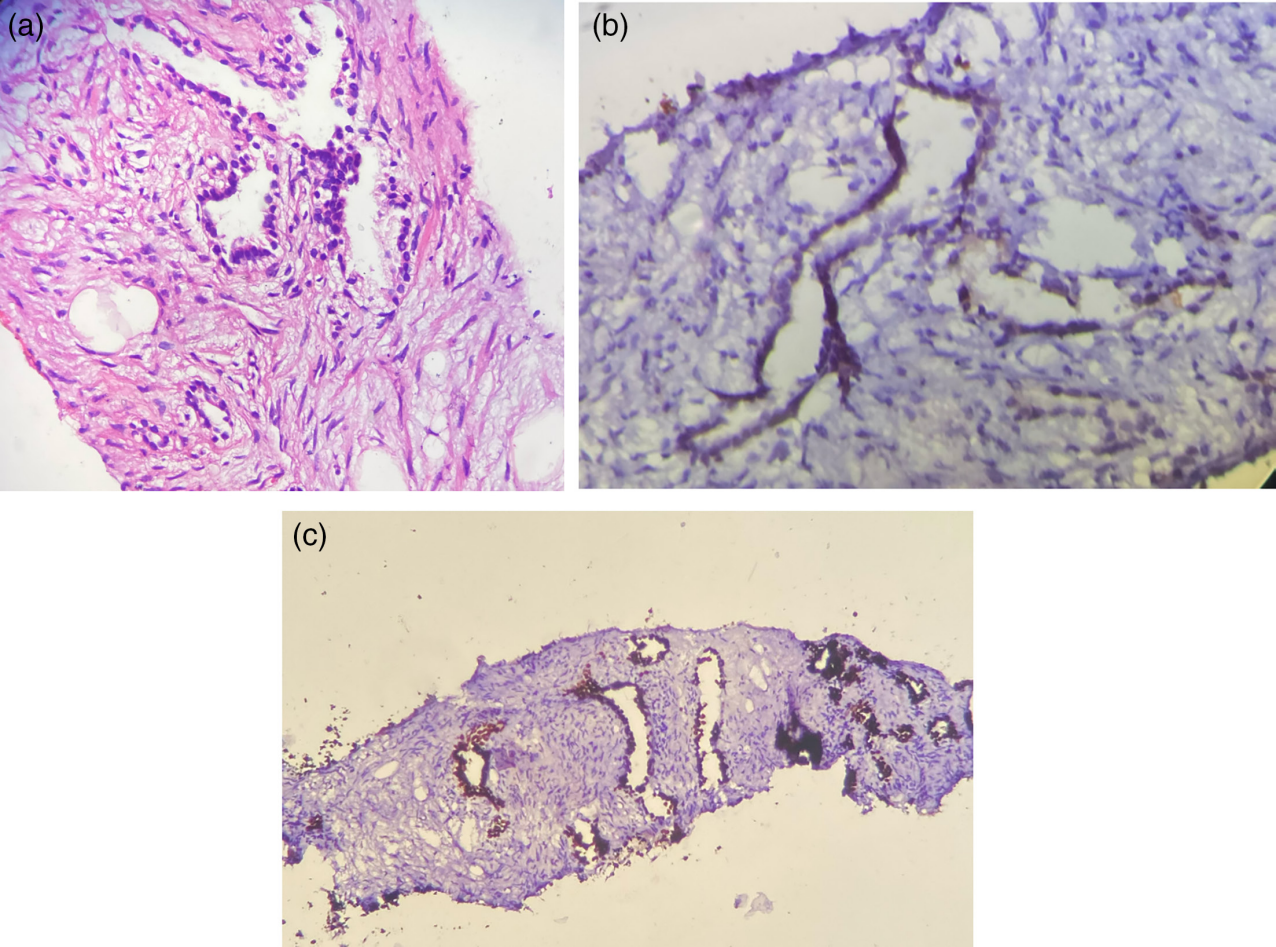
(a) Histopathologic section of the resected lesion stained with Haematoxylin & Eosin (H&E), demonstrating cartilage islands and fibromyxoid tissue, consistent with pulmonary hamartoma. (b) IHC staining for Napsin A, showing absence of cytoplasmic positivity, supporting the benign nature of the lesion and excluding adenocarcinoma. (c) Immunohistochemical (IHC) staining for TTF‐1 in the tumour specimen, negative for nuclear expression, ruling out primary lung adenocarcinoma.

To clarify the nature of the upper lobe lesion, a CT‐guided core needle biopsy was performed. The initial pathological report indicated adenocarcinoma, supported by immunohistochemical staining positive for CK7, TTF‐1, and Napsin‐A, and negative for CK20, CDX‐2, GATA‐3, and PAX‐8 (Figure 2).

Further staging with PET‐CT scan showed mild FDG uptake confined to the right upper lobe lesion without evidence of extrathoracic metastasis.

For definitive diagnosis and treatment, the patient underwent video‐assisted thoracoscopic surgical excisional biopsy of the mass. Intraoperative frozen section analysis excluded adenocarcinoma and suggested a chondrogenic lesion consistent with hamartoma or teratoma. Surgical margins were safe, and therefore, the surgery was concluded without performing lobectomy or segmentectomy. Final histopathology confirmed the diagnosis of pulmonary hamartoma.

Postoperatively, the patient had an uneventful recovery with complete resolution of her symptoms and remained well during follow‐up.

## 3 Discussion

This case report highlights the diagnostic challenges of pulmonary hamartomas due to their overlapping clinical, radiological, and histopathological features with malignant lung tumours such as adenocarcinoma. Pulmonary hamartomas (PH) are the most common benign lung neoplasms, typically composed of mixed mesenchymal tissues including cartilage, fat, and connective tissue. They are frequently discovered incidentally on imaging studies and tend to be asymptomatic in most cases. However, in rare presentations such as ours—where the patient presented with hemoptysis—these lesions can produce respiratory symptoms when located near central airways, necessitating differentiation from malignant tumours like adenocarcinoma [1, 4, 6, 7].

Although peripheral pulmonary hamartomas generally do not cause hemoptysis, rare cases of haemorrhage have been reported. The mechanism of hemoptysis in our case is likely related to mechanical irritation or inflammatory changes in the adjacent bronchial mucosa caused by the tumour's proximity, even without direct invasion or perforation of the bronchial wall. A similar phenomenon has been described in the literature, such as in a reported case where bleeding from a peripheral hamartoma reached a non‐adjacent bronchus via the pulmonary artery sheath, resolving after tumour removal. Thus, mechanical and inflammatory irritation by hamartomas located near airways may provoke hemoptysis, which typically resolves following surgical excision [8].

Core needle biopsy (CNB) is a minimally invasive diagnostic method commonly used for lung lesions. Despite its clinical utility, CNB may result in misdiagnosis due to limited tissue sampling, particularly when pathological features of hamartomas overlap with malignancies. Immunohistochemical findings, although helpful, can sometimes be misleading in small samples, leading to diagnostic errors as demonstrated in our case [5, 9]. The immunohistochemical profile of the case is summarised in Table 1.

**TABLE 1:** Immunohistochemistry (IHC) results in the current case and key markers for differential diagnosis.

Marker	Result in current case	Typical positivity in adenocarcinoma	Notes on diagnostic significance
CK7	Positive	Positive	Common marker of lung adenocarcinoma epithelium
TTF‐1	Positive	Positive	Nuclear marker of lung adenocarcinoma and benign epithelium
Napsin‐A	Positive	Positive	Cytoplasmic marker related to adenocarcinoma
CK20	Negative	Negative	Usually negative in primary lung adenocarcinoma
CDX‐2	Negative	Negative	Marker for gastrointestinal origin tumours
GATA‐3	Negative	Negative	Marker for breast and urothelial carcinomas
PAX‐8	Negative	Negative	Marker for thyroid and renal tumours

Immunohistochemistry (IHC) is a valuable diagnostic tool; however, it has limitations. Markers such as Napsin A and TTF‐1, typically indicative of pulmonary adenocarcinoma, may show positivity due to the presence of entrapped benign respiratory epithelium within pulmonary hamartomas, rather than true malignant expression. Such false‐positive staining results can lead to diagnostic pitfalls, especially in small biopsy specimens, underscoring the critical importance of correlating IHC results with histopathological, radiological, and clinical findings. Hence, definitive diagnosis usually requires examination of the entire resected specimen [10, 11].

Reviewing similar cases in the literature (Table 2) revealed consistent diagnostic difficulties, with several reports documenting misdiagnosis of pulmonary hamartomas as adenocarcinoma based on limited biopsy specimens [4, 5, 6]. These findings emphasise the importance of comprehensive evaluation and a cautious approach when biopsy results do not correlate with imaging or clinical presentation.

**TABLE 2:** Summary of reported cases of pulmonary hamartoma initially misdiagnosed as adenocarcinoma.

Author(s)	Number of cases	IHC profile (key markers)	Initial diagnosis	Final diagnosis	Definitive diagnosis method
Kış et al.	5	TTF‐1+, Napsin‐A+, CK7+	Adenocarcinoma suspected	Pulmonary hamartoma	Surgical resection
Li et al.	3	Similar markers to adenocarcinoma	Lung cancer suspected	Pulmonary hamartoma	Surgical pathology
Chatzopoulos et al.	7	Variable, occasional TTF‐1 positivity	Malignancy suspected	Pulmonary hamartoma	Histopathology after resection

Radiological imaging, especially computed tomography (CT), plays an important role in identifying characteristic features of pulmonary hamartomas such as intralesional fat and popcorn‐like calcifications, which are present in up to 60% and 25%–50% of cases, respectively. However, the absence of these features reduces the specificity of diagnosis and necessitates histopathological confirmation [12].

However, not all pulmonary hamartomas contain enough fat to be reliably detected by CT alone. Chemical‐shift MRI, which provides higher sensitivity and specificity for fat detection, can be an important complementary imaging modality in cases with inconclusive CT findings. Studies have demonstrated that chemical‐shift MRI can accurately identify intranodular fat in pulmonary hamartomas, improving diagnostic confidence and aiding in differentiation from malignant lesions [13].

Surgical resection is considered the gold standard for definitive diagnosis and treatment, allowing complete pathological evaluation and lesion removal. Lung‐sparing procedures such as wedge resection or enucleation are preferred when feasible, while lobectomy may be warranted for larger, centrally located, or radiologically suspicious lesions. Frozen section intraoperative consultation assists in distinguishing benign from malignant lesions during surgery [14, 15]. Avoiding misdiagnosis is crucial to prevent unnecessary aggressive treatments that carry significant morbidity. Multidisciplinary collaboration among pulmonologists, radiologists, pathologists, and thoracic surgeons optimises the diagnostic process and patient outcomes [15, 16]. Limitations of this study include reliance on case reports in the literature and a lack of larger cohort analyses to generalise findings. Future studies incorporating molecular diagnostic markers and advanced imaging techniques may improve preoperative diagnostic accuracy. Pulmonary hamartomas present significant diagnostic challenges due to their clinical, radiological, and histopathological resemblance to malignant lung tumours such as adenocarcinoma. Small biopsy specimens and limited immunohistochemical panels can lead to false‐positive findings and misdiagnosis. Therefore, comprehensive evaluation that integrates clinical, radiological, and pathological data is essential. Surgical resection remains the gold standard for definitive diagnosis, particularly in cases with discordant findings. Awareness of these diagnostic pitfalls can prevent unnecessary aggressive treatments and improve patient outcomes.

## Author Contributions

M.M.F. and E.H. collected clinical data and wrote the manuscript. M.B.S. performed the surgery and contributed to the manuscript. R.S. revised the manuscript. All authors read and approved the final manuscript.

## Funding

The authors have nothing to report.

## Consent

Written informed consent for publication of this manuscript and accompanying images was obtained from the patient, and the form used complies with the Journal's author guidelines.

## Conflicts of Interest

The authors declare no conflicts of interest.

## Data Availability

The data that support the findings of this study are available on request from the corresponding author. The data are not publicly available due to privacy or ethical restrictions.
